# Biomarker development for the tau therapeutic landscape

**DOI:** 10.1002/alz70856_099835

**Published:** 2025-12-24

**Authors:** Danielle Graham

**Affiliations:** ^1^ Biogen, Cambridge, MA, USA

## Abstract

The hallmark neuropathological changes associated with Alzheimer's disease (AD) include extracellular amyloid beta plaques and intracellular neurofibrillary tangles (NFTs) composed of hyperphosphorylated tau. Because NFTs are strongly associated with clinical impairment, multiple therapeutic programs focused on reducing tau pathology have been initiated, necessitating the development of biomarkers specific to tau pathology. Current tau blood‐based biomarkers (e.g., phosphorylated tau 181 [p‐tau181] and 217 [p‐tau217]) are more closely associated with amyloid pathology than tau pathology and may not allow specific assessment of the presence of, or therapeutic impact on, neuropathological tau. BIIB080 is a microtubule associated protein tau (*MAPT*)–targeting antisense oligonucleotide that reduces production of tau protein. Exploratory analysis from the BIIB080 Phase 1b trial (NCT03186989) demonstrated that BIIB080 treatment was associated with a dose‐dependent and sustained reduction in soluble tau protein in cerebrospinal fluid (CSF), and exploratory analysis evaluating the change in soluble *p*‐tau in plasma is in progress. Moreover, BIIB080 impacted aggregated parenchymal tau pathology, as measured by tau positron emission tomography, demonstrating a reduction of tau pathology from baseline across all brain composites assessed at the end of the long‐term extension. These data were the first to show this magnitude of treatment response on this pathological hallmark of AD. The ongoing Phase 2 CELIA trial (NCT05399888) will further evaluate impacts of BIIB080 on these biomarkers, as well as the safety and efficacy of BIIB080 in a larger and broader AD population. Emerging candidates for tau‐specific biomarkers include *p*‐tau205 and a portion of the microtubule binding region of tau containing the residue 243 (MTBR‐tau243). Evaluation of the impact of BIIB080 on these measures and other novel biomarkers of tau pathology is ongoing. However, these biomarkers are currently measured in CSF only, and evidence for their use as treatment‐response measures is limited. Based on emerging tau biomarker advances and biomarker results from BIIB080, this presentation will provide future directions on the use and importance of tau biomarkers for development of therapies targeting tau.

